# Problematic smartphone use is more strongly associated with academic procrastination than self-reported daily use duration

**DOI:** 10.3389/fpsyg.2026.1963771

**Published:** 2026-10-28

**Authors:** Lucica Emilia Coșa, Irina Mihaela Trifan

**Affiliations:** 1 Department of Teacher Training, George Emil Palade University of Medicine, Pharmacy, Science, and Technology of Targu Mures, Targu Mures, Romania; 2 Department of Sciences and Letters 2, Faculty of Sciences and Letters “Petru Maior”, George Emil Palade University of Medicine, Pharmacy, Science, and Technology of Targu Mures, Targu Mures, Romania

**Keywords:** academic procrastination, daily smartphone use, educational stage, problematic smartphone use, self-efficacy, self-regulation

## Abstract

**Introduction:**

Problematic smartphone use has been associated with academic procrastination, but it remains unclear whether this association reflects the amount of time spent using a smartphone or difficulty regulating its use. This cross-sectional study compared problematic smartphone use with self-reported daily use duration as predictors of academic procrastination. It also, examined general self-efficacy as a candidate intervening variable and educational stage as an exploratory moderator, and provided a preliminary psychometric evaluation of a new 27-item measure of problematic smartphone use.

**Methods:**

A convenience sample of 973 Romanian adults enrolled in secondary or higher education completed measures of problematic smartphone use, self-reported daily use duration, general self-efficacy, and academic procrastination. All participants were at least 18 years old, so the secondary-education subgroup comprised adult students in their final high-school years. Analyses included competing confirmatory factor models, hierarchical regression, comparison of dependent correlations, a bootstrap indirect-effect model, and exploratory moderation.

**Results:**

Problematic smartphone use was positively associated with academic procrastination (*r* = 0.21, 95% CI [0.15, 0.27], *p* < 0.001), whereas self-reported daily use duration was not (*r* = 0.04, *p* = 0.192). The two dependent correlations differed significantly (Williams test, *p* < 0.001), and problematic smartphone use explained additional variance beyond self-reported use duration (Δ*R*^2^ = 0.042). The association remained similar after removing items conceptually adjacent to procrastination, although conceptual overlap cannot be excluded. General self-efficacy did not account for the association in the hypothesized direction. In exploratory analyses, the association was stronger in the secondary-education subgroup (*r* = 0.31) than among university students (*r* = 0.15; interaction *p* = 0.016, Δ*R*^2^ = 0.007).

**Discussion:**

Academic procrastination was more consistently associated with problematic smartphone use than with self-reported daily use duration, although the explained variance was small (*R*^2^ = 0.04). Duration was self-reported with a single item, so the comparison does not extend to objectively recorded screen time. The findings are consistent with a self-regulation account; however, the cross-sectional design precludes causal or temporal inferences. Both measures require independent validation, and the findings should be replicated with independently validated instruments.

## 1 Introduction

Smartphones have become part of everyday routine for most people and serve both educational and recreational functions for those in formal education. Because the device combines permanent availability with variable rewards obtained immediately, it competes strongly for task-directed attention (Kushlev et al., 2016; Su et al., 2021). However, a growing body of research suggests that the relevant problem is not the device itself but the way it is used. The distinction between the quantity and problematic quality of smartphone use is the central concern of the present study.

Academic procrastination is defined as the voluntary postponement of an academic activity despite awareness of its negative consequences (Solomon and Rothblum, 1984; Tice and Baumeister, 1997; Steel, 2007). It is widespread among students and is associated with weaker academic performance, elevated stress, and reduced well-being (Tice and Baumeister, 1997; Steel, 2007), and beyond the academic domain with adverse health outcomes (Sirois et al., 2003; Sirois and Pychyl, 2013). It has also been characterized as an irrational delay accompanied by anxiety and guilt (Schraw et al., 2007; Steel, 2010).

Social cognitive theory provides the framework adopted here. Self-regulation is the process through which individuals monitor, evaluate, and adjust their own behavior in relation to goals and anticipated consequences (Bandura, 1986), while self-efficacy is a malleable belief that develops through experience, particularly in challenging situations requiring sustained effort (Coșa and Cernat, 2025). In education, Zimmerman (2000, 2002) describes self-regulated learning as the continuous planning, monitoring, and evaluation of one’s own cognitive, motivational, and behavioral strategies, organized in a cycle of forethought, performance, and self-reflection. From this perspective, academic procrastination can be understood as a dysfunction of that cycle: goals are set but the behaviors required to reach them are postponed, self-monitoring does not lead to effective adjustment, and self-reflection is frequently accompanied by guilt and self-blame rather than strategy revision (Zimmerman, 2002; Wolters, 2003; Steel, 2007).

Three further accounts are compatible with this framework and are noted here without being tested. Temporal motivation theory holds that the decision to delay depends on expectancy, task value, impulsiveness, and delay to reward; thus, academic tasks with distant benefits tend to be displaced by immediately rewarding activities (Steel, 2007; Steel and König, 2006). The mood-regulation account treats procrastination as short-term avoidance of the negative affect elicited by aversive tasks, prioritizing immediate emotional state over long-term goals (Baumeister and Heatherton, 1996; Sirois and Pychyl, 2013). Self-determination theory locates the quality of motivation at the basis of self-regulation and links procrastination to reduced autonomous motivation and increased amotivation (Deci and Ryan, 1985; Katz et al., 2014; Ryan and Deci, 2000); from this angle, problematic smartphone use may reflect a compensatory strategy associated with need frustration rather than an autonomous choice (Billieux et al., 2015; van Deursen et al., 2015).

Empirically, problematic smartphone use has been associated with higher levels of academic procrastination (Lepp et al., 2014; Samaha and Hawi, 2016; Walsh et al., 2010) and immediate access to social networks, games, and messaging applications favors the reorientation of attention toward immediate rewards at the expense of academic tasks (Kushlev et al., 2016; Rosen et al., 2011). Phone-related delay frequently takes the form of cyberloafing, that is, non-academic internet use during time allocated to study (Lim, 2002; Vitak et al., 2011), which interrupts learning and affects both attention maintenance and information retention (Junco, 2012; Wood et al., 2012). A recent meta-analysis confirmed a moderate and robust association between problematic smartphone use and academic procrastination (Chen and Lyu, 2024).

Evidence also indicates that duration of use and problematic use are distinct dimensions with different associations with psychological and academic outcomes: time spent on the phone is an inconsistent predictor of maladjustment, whereas problematic use is consistently associated with negative outcomes (Billieux et al., 2015; Panova and Carbonell, 2018). One mechanism that may account for this asymmetry is the difficulty in terminating an activity once initiated. The I-PACE model explains this difficulty through the interaction of affective state, reactivity to reward-related cues, and reduced inhibitory control (Brand et al., 2019), an account supported by evidence of inhibitory-control deficits among users with high levels of problematic use (Chen et al., 2016), by neuroimaging findings in networks involved in cognitive control and reward processing (Horvath et al., 2020), and by network analyses placing impulsivity and emotion-regulation difficulties at the center of problematic smartphone use (Cho and Kim, 2025). Application design may also contribute: short-video feeds with infinite scrolling provide a continuous stream of content and few natural stopping points (Su et al., 2021).

Self-efficacy is the belief that one can organize and carry out actions required to reach a goal (Bandura, 1997). Higher levels are associated with initiating and sustaining academic activities, whereas lower levels are correlated with procrastination (Klassen et al., 2008; Pajares, 1996; Schunk, 1995; Steel, 2007). Repeated difficulty in controlling smartphone use may plausibly diminish self-efficacy, which in turn may favor the postponement of tasks perceived as difficult. The present study assessed general rather than domain-specific self-efficacy, and the results were interpreted accordingly.

Two gaps in the current literature motivated this study. First, although the association between problematic smartphone use and procrastination is well documented, few studies have measured problematic use and use duration simultaneously in the same sample to test directly which of the two is associated with academic delay. Second, candidate mechanisms remain disputed: domain-specific self-efficacy appears to intervene in some samples (Li et al., 2020; Liu et al., 2022; Zhao et al., 2025), but the role of generalized self-efficacy remains unclear.

This study aimed to examine whether academic procrastination is more closely associated with problematic smartphone use than with the self-reported duration of use. The conceptual model, hypotheses, and exploratory research questions are summarized in Figure 1. Three primary hypotheses were formulated: (H1) problematic smartphone use is positively associated with academic procrastination; (H2) self-reported daily use duration shows a weaker association with procrastination than problematic use; and (H3) general self-efficacy statistically accounts for part of the association between problematic smartphone use and academic procrastination. An exploratory research question (RQ1) concerned the possible differences associated with educational stages in the relationship between problematic smartphone use and academic procrastination. Because no validated Romanian measure covering contemporary manifestations of the construct was available, problematic use was assessed using an instrument constructed for this study, the MSUPS, whose internal properties are reported here so that the main analyses can be interpreted. This study was not a scale-validation study and did not present the MSUPS as validated. Because eligibility required a minimum age of 18 years, the secondary education subsample comprised adult students in their final high school years and was not representative of high school students or adolescents in general; all results involving this subgroup are interpreted in that light.

**Figure 1:**
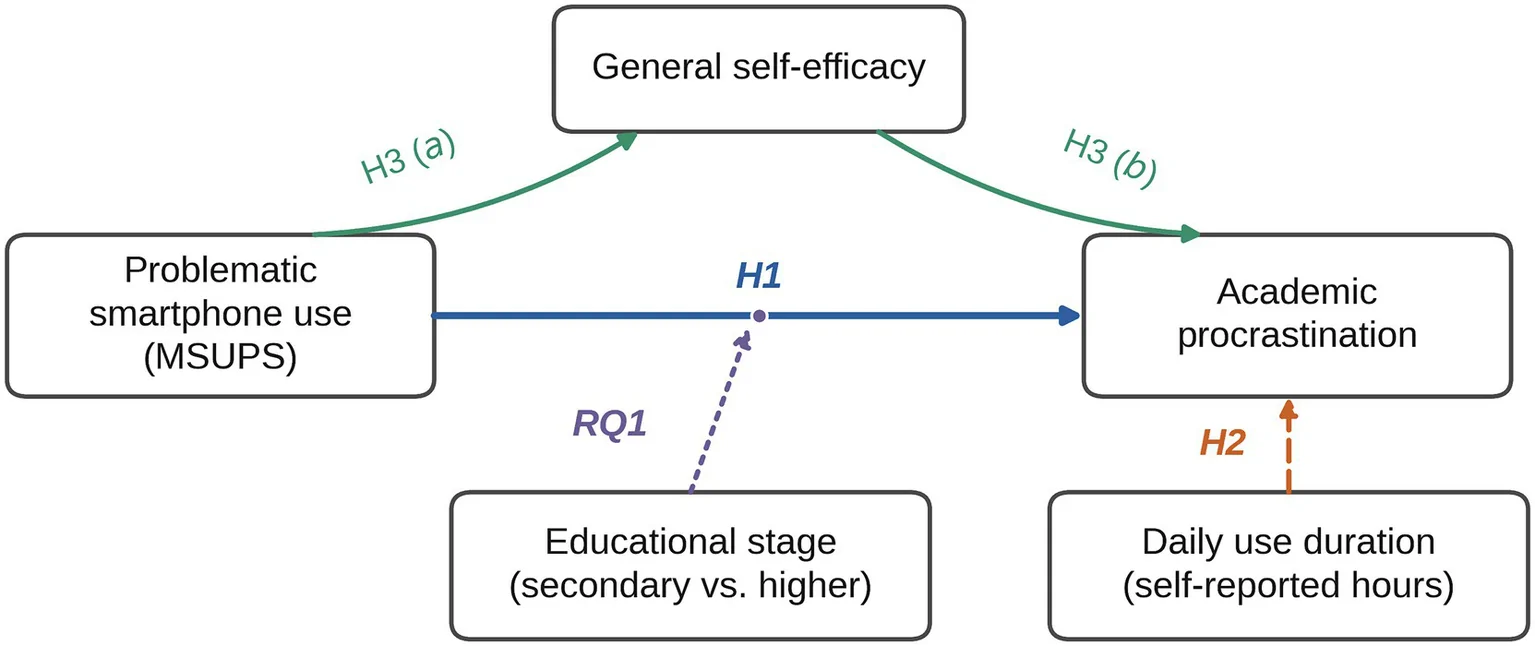
Conceptual model of the study. The solid path represents the hypothesized association between problematic smartphone use and academic procrastination (H1); the curved paths through general self-efficacy represent the hypothesized indirect association, with a negative path a and a positive path b (H3); the dashed path represents self-reported daily use duration as a competing predictor (H2); and the dotted path represents the exploratory moderation by educational stage (RQ1). Given the cross-sectional design, the paths do not imply causal or temporal ordering, and paths a and b denote a statistical decomposition rather than mediation. MSUPS, Multidimensional Smartphone Use Problem Scale.

## 2 Materials and methods

### 2.1 Study design

This study used a cross-sectional correlational design to examine the relationships between problematic smartphone use, self-reported daily use duration, general self-efficacy, educational stage, and academic procrastination. Data were collected through an online questionnaire and analyzed using descriptive statistics, reliability analyses, competing confirmatory factor models, a split-sample internal evaluation of the factor structure, measurement invariance testing, correlations, hierarchical regression, a bootstrap indirect-effect model, and exploratory moderation analysis followed by simple-slope probing. The design permits the assessment of associations between variables but cannot establish the direction of those relationships or causal effects.

### 2.2 Eligibility criteria and data screening

The inclusion criteria were as follows: (1) enrolment in a Romanian secondary or higher education institution at the time of data collection; (2) use of a personal smartphone; (3) age of at least 18 years; and (4) provision of informed consent before completing the questionnaire.

The exclusion criteria, defined before any statistical analysis, were duplicate records and responses that failed the pre-specified data-quality rule. Six records were identified in which the consent text had been recorded twice within the same submission, the signature of a resubmitted form; the first of these was retained and the remaining five were removed, one of which also showed invariant responding across all 27 MSUPS items. Therefore, the criterion applied was the repeated recording of consent within a single record and not the identity of response vectors: the six records were not exact duplicates of one another.

### 2.3 Participants and procedure

The study included 973 participants recruited through an online questionnaire distributed via the communication channels of educational institutions and via social networks. Participation was voluntary and unrewarded. Of the 978 responses collected, five were excluded under the data-quality rule described above, yielding a final sample of *N* = 973. Analyses involving age were conducted on 971 cases because two responses to the age item were invalid.

The sample comprised 342 participants enrolled in secondary education (35.1%) and 631 enrolled in higher education (64.9%). All participants were aged at least 18 years. Age was collected in categories and recoded into representative values for secondary analyses only. With respect to gender, 667 participants (68.6%) identified as female, 296 (30.4%) as male, and 10 selected other options or multiple gender categories.

Data were collected between 14 January and 11 June 2026 using Google Forms. Participants received an information sheet and provided electronic informed consent before accessing the questionnaire (Supplementary Table S4); only those who consented could proceed. The study was approved by the Research Ethics Committee of the George Emil Palade University of Medicine, Pharmacy, Science, and Technology of Târgu Mureș, Romania (decision no. 3822 of 17 June 2025). All items of the main scales were configured as mandatory; therefore, there were no missing values on these measures. Because the questionnaire was distributed through institutional channels and social networks, a participation rate could not be calculated.

### 2.4 Measures

#### 2.4.1 Problematic smartphone use

Problematic smartphone use was assessed using the Multidimensional Smartphone Use Problem Scale (MSUPS), a 27-item self-report instrument developed by the authors for the present study using a deductive, content-domain-centered approach. Items were written in Romanian after examination of the conceptual definitions and item content of established measures of problematic mobile phone use (Agus et al., 2022; Bianchi and Phillips, 2005; Kwon et al., 2013). The wordings were composed for the present instrument and did not constitute a direct translation or adaptation of an existing scale. Two psychologists rated the relevance, clarity, and representativeness of each item with respect to its intended content domain; their comments were reviewed by the authors, and item wordings and domain allocations were revised by consensus before data collection.

Five content domains were defined *a priori*, before data collection, by deductively mapping the manifestations described in existing models and instruments (Agus et al., 2022; Bianchi and Phillips, 2005; Griffiths, 2005; Kwon et al., 2013): (1) compulsive use and loss of control, covering unintended use, difficulty reducing use, and low resistance to the urge to check (six items); (2) psychological dependence, including persistent preoccupation and discomfort in the absence of the smartphone (seven items); (3) functional interference, reflected in effects on sleep, productivity, responsibilities, and academic or occupational performance (six items); (4) impairment of social functioning, covering conflict with or reduced interaction with close others (four items); and (5) compensatory use, defined as using the smartphone to avoid tasks, problems, or unpleasant affective states (four items). Item allocation to domains was reviewed by the two psychologists and finalized by consensus with the authors of this study. This structure represents the content development framework and not the outcome of an exploratory factor analysis; the initial allocation is reported in Supplementary Table S1.

Items were rated on a five-point Likert scale (1 = not at all, 5 = always) with reference to habitual smartphone use, without a pre-specified time frame. The global score was computed as the mean of the 27 items, with higher scores indicating higher levels of problematic smartphone use. All items were worded in the same direction; there were no reverse scored items. The factor structure of this initial version was empirically examined in the present sample (Section 3.3). The study hypotheses were tested using the global score, and the psychometric justification for that decision, together with its limitations, is presented in Sections 3.3 and 4.

For use in international research, the Romanian version was translated into English, and the resulting version was independently back-translated into Romanian in accordance with the recommendations of the International Test Commission (2017). The back-translated version was compared with the original wordings, and semantic discrepancies were resolved by consensus, with conceptual equivalence rather than literal translation as the criterion. The English version is provided in the Supplementary material. Because it was not administered to participants, its psychometric properties were not evaluated. The MSUPS was used for research purposes only and, at this stage, has no norms or clinical cut-offs; it is intended for dimensional assessment in research and not for diagnosis or case identification.

#### 2.4.2 General self-efficacy

Generalized self-efficacy was measured using the 10-item General Self-Efficacy Scale (Schwarzer and Jerusalem, 1995; e.g., “I can always manage to solve difficult problems if I try hard enough”), rated from 1 (not at all true) to 4 (exactly true) and averaged. Internal consistency in the present sample was good (*α* = 0.89; ω = 0.89; corrected item–total correlations 0.55–0.71). A Romanian adaptation with normative data is available (Vasiliu et al., 2015).

#### 2.4.3 Academic procrastination

Academic procrastination was assessed using a short Romanian index of six items adapted conceptually from the Procrastination Assessment Scale for Students (PASS; Solomon and Rothblum, 1984). The items assessed the frequency of postponing academic tasks and were rated on a five-point Likert scale (1 = never, 5 = always). The score used in the analyses was the mean of the six items, with higher values indicating higher levels of academic procrastination. Internal consistency was acceptable (*α* = 0.80). Full item wording in Romanian and English, together with per-item descriptive statistics and corrected item–total correlations, is provided in Supplementary Table S2. Because the instrument is an unvalidated conceptual adaptation of PASS rather than a translation of the original scale, and because it constitutes the primary criterion of the study, its properties were examined explicitly, including a one-factor confirmatory model (Section 3.4). It is treated throughout as a composite index of delay frequency and not as a validated version of PASS.

Perceived reasons for procrastination were assessed separately using eight items covering the principal reasons for delay (e.g., perfectionism, fear of failure, lack of interest, organizational difficulties, and distractibility), rated from 1 (not at all true) to 5 (very true). These items were analyzed exploratively and separately from hypothesis testing.

#### 2.4.4 Daily smartphone use and sociodemographic variables

Average daily smartphone use duration was assessed using a single item comprising six ordinal categories: “less than 1 h” to “more than 5 h.” For parametric analyses, the categories were recoded using interval midpoints (0.5–5.5 h). Because the highest category was open-ended and contained a large proportion of participants (36.0%), sensitivity analyses using alternative operationalizations of the variable were conducted (Section 3.8).

Sociodemographic data were also collected on sex, educational institution, year of study, age category, sibling position, and age at first smartphone ownership (Supplementary Table S5). The original age response categories and the representative value assigned to each are reported in Supplementary Table S4.

### 2.5 Derivation of educational stage

Educational stage was derived from the year-of-study item, which allowed the selection of a university year (I–V) or an option for high-school students. The coding rule was as follows: a participant was classified as secondary education (coded 1) if the response contained a marker such as high school, irrespective of graphical form (capitalization, diacritics, spaces, or additional punctuation), and as higher education (coded 0) if the response contained only a year of study expressed in Roman numerals. For the 13 cases in which markers of both types were selected, priority was given to the secondary-education marker; the sensitivity of the results to this decision is reported in Section 3.10. The rule classified all 973 participants with no missing values, yielding 342 participants in secondary and 631 in higher education.

### 2.6 Data analysis

Substantive analyses were conducted using IBM SPSS Statistics 23 with the PROCESS macro (Hayes, 2022), using HC3 heteroscedasticity-robust standard errors and percentile bootstrap with 5,000 resamples for indirect effects. Factor analyses were conducted in R 4.5.2 with the packages lavaan 0.7–2 (Rosseel, 2012), psych 2.6.5, semTools 0.5–9, and polycor. Because the MSUPS items are ordinal with five response categories, all factor models were estimated using polychoric correlations by diagonally weighted least squares with mean- and variance-adjusted test statistics (WLSMV; delta parameterization). A two-sided significance level of *α* = 0.05 was adopted throughout the study. Scale scores were computed as the mean of the item responses, preserving the original Likert metric.

The initial psychometric evaluation of the MSUPS proceeded in four steps. First, the reliability of the five *a priori* domains and of the total score was estimated using Cronbach’s α, McDonald’s ω, and corrected item–total correlations. Second, the *a priori* structure was tested confirmatorily against three competing models: a one-factor model, a correlated five-factor model, a second-order model in which the five domains loaded on a general factor, and a bifactor model in which each item loaded simultaneously on a general factor and on its domain-specific factor. Model fit was evaluated using CFI, TLI, RMSEA, and SRMR against the conventional criteria CFI and TLI ≥ 0.95 and RMSEA and SRMR ≤ 0.08 (Hu and Bentler, 2009), and the proportion of reliable total-score variance attributable to the general factor was estimated with hierarchical omega (ωH) from the bifactor solution (Reise, 2012). Residual correlations of the bifactor model were inspected to identify sources of misfit. Third, to reduce the circularity involved in developing, evaluating, and applying the instrument within one sample, a split-sample internal evaluation was conducted by randomly splitting the sample into an exploratory half (*n* = 486) and a second half (*n* = 487). In the exploratory half, the number of factors was determined by parallel analysis (Horn, 2025; O’Connor, 2000), followed by exploratory factor analysis with oblique rotation; the replicability of the loading configuration was assessed in the second half using Tucker congruence coefficients (Lorenzo-Seva and ten Berge, 2006), and the competing simple-structure models were re-estimated there together with an exploratory three-factor model allowing cross-loadings. Fourth, measurement invariance across educational stages was examined in two complementary ways. An initial analysis under continuous treatment of items followed the configural–metric–scalar sequence, using likelihood-ratio difference tests and the criterion ΔCFI ≤ 0.01 (Chen, 2007). Because that approach is inadequate for ordinal items, invariance was re-examined on polychoric correlations following the sequence recommended for ordered-categorical data: configural model, equality of thresholds, and equality of thresholds and loadings (Wu and Estabrook, 2025). Threshold equality was tested by a Wald test on the 108 thresholds estimated independently in the two groups, using their full asymptotic covariance matrix, and loading equality by comparing the bifactor model with free versus constrained loadings. Invariance conclusions were interpreted conditionally on the fit of the baseline model.

Pearson correlations were reported with 95% confidence intervals obtained by Fisher’s *z* transformation, and Spearman coefficients were computed as sensitivity checks. Effect-size magnitudes were interpreted following Cohen (1988). H1 was evaluated using correlation and simple regression. H2 was tested using hierarchical regression with both predictor entry orders and a formal comparison of the two dependent correlations with academic procrastination using the Williams test (Steiger, 1980). H3 was examined with an indirect-effect model specifying problematic smartphone use as the independent variable, general self-efficacy as the intervening variable, and academic procrastination as the outcome. This model decomposes an observed association in cross-sectional data and does not test temporal mediation. RQ1 was examined exploratorily with a regression model including the interaction between problematic smartphone use and educational stage (0 = higher education, 1 = secondary education), with the continuous predictor mean-centered, followed by simple-slope analysis; the difference between the two group correlations was tested by Fisher’s *z* transformation. Regression assumptions were checked by examining multicollinearity, independence, distribution of residuals, heteroscedasticity, and influential observations.

Robustness was assessed through a series of sensitivity analyses. The association between use duration and academic procrastination was additionally examined using Spearman ‘s correlation on the original ordinal categories, the Kruskal–Wallis test, and repetition of the analysis after excluding the open-ended upper category. The influence of measurement error in the single-item duration measure was examined by correcting for attenuation under several assumed reliability values. Potential content overlap between the MSUPS and the academic procrastination index was assessed by recomputing the MSUPS score after removing the items referring directly to distraction, task avoidance, productivity, and academic interference. Common method variance was examined descriptively using Harman’s single-factor test (Podsakoff et al., 2003). The main association was also re-estimated after excluding respondents with invariant responding on the general self-efficacy scale, separately by gender, and after removing the single item worded from the perspective of others’ perceptions. The robustness of the educational-stage interaction was assessed through four analyses varying the coding decisions: (a) reclassifying the multiply marked cases as higher education; (b) excluding those cases; (c) excluding participants with indications of post-secondary non-tertiary education, who fall into neither the high-school nor the university category; and (d) the independent recoding based on the institution free-text field. The interaction was additionally re-estimated separately in the female and male subsamples. Perceived reasons for procrastination were analyzed separately and exploratorily.

The sample size was not determined by an *a priori* power analysis; all eligible participants recruited during the data collection window were included. At *N* = 973, *α* = 0.05, and 80% power, the minimum detectable effect was approximately *r* = 0.09 for correlations and Δ*R*^2^ = 0.008 for the interaction term (Cohen, 1988). The study and the analysis plan were not preregistered. H1–H3 were formulated *a priori* and are treated as primary hypotheses, whereas RQ1 and the analyses of procrastination reasons were exploratory. A further *post hoc* analysis of the association between age at first mobile phone ownership and current daily smartphone use is reported in Supplementary Table S6; it was added after the initial analyses were completed and was not adjusted for multiple comparisons.

## 3 Result

Sections 3.1 and 3.2 report descriptive statistics and reliability; Sections 3.3 to 3.5 report the psychometric analyses required to justify the scores used and the assumption checks; Sections 3.6 to 3.9 report the confirmatory tests of H1 to H3; Section 3.10 reports the exploratory moderation (RQ1); and Sections 3.11 to 3.13 report the common-method check, the sensitivity analyses and the exploratory analysis of self-reported reasons for procrastination. *Post hoc* analyses are reported in Supplementary Table S6.

### 3.1 Descriptive statistics and reliability

Descriptive statistics and reliability coefficients are reported in Table 1. The distributions of the main variables showed only modest departures from normality (|skewness| ≤ 0.70; |kurtosis| ≤ 0.62). The mean problematic smartphone use score was 2.62 (SD = 0.76) and the mean academic procrastination score was 3.02 (SD = 0.94), both on scales ranging from 1 to 5. For daily use duration, 36.0% of participants selected the open-ended upper category (“more than 5 h”) and 60.2% fell in the two highest response categories.

**Table 1:** Descriptive statistics and internal consistency of the study variables (*N* = 973).

Variable	*M*	SD	Min	Max	Skewness	Kurtosis	*α*	*ω*
Problematic smartphone use (MSUPS; 1–5)	2.62	0.76	1.00	5.00	0.34	−0.08	0.94	0.94
General self-efficacy (GSES; 1–4)	3.09	0.57	1.00	4.00	−0.53	0.38	0.89	0.89
Academic procrastination (1–5)	3.02	0.94	1.00	5.00	−0.01	−0.62	0.80	0.80
Self-reported daily smartphone use (0.5–5.5 h)	4.24	1.22	0.50	5.50	−0.70	−0.27	—	—

MSUPS, Multidimensional Smartphone Use Problem Scale; GSES, General Self-Efficacy Scale; *M*, mean; SD, standard deviation; *α*, Cronbach’s alpha; *ω*, McDonald’s omega. Scale scores were computed as the mean of the item responses and retain the original Likert metric. Coefficients of internal consistency are not applicable to daily smartphone use, which was assessed with a single item recoded to category midpoints.

The MSUPS showed high internal consistency (*α* = 0.941; ω = 0.942; corrected item–total correlations 0.42–0.75). The General Self-Efficacy Scale showed good reliability (*α* = 0.89; corrected item–total correlations 0.55–0.71). The academic procrastination index showed acceptable reliability (*α* = 0.80; corrected item–total correlations 0.27–0.67). The item with the lowest corrected item–total correlation (0.27; reading the recommended bibliography) was retained for the reasons set out in Section 2.4.3. The properties of this scale are examined in Section 3.4.

### 3.2 MSUPS domain reliability and intercorrelations

The correlation matrix of the 27 MSUPS items was suitable for factor analysis (KMO = 0.96; per-item KMO ≥ 0.93; Bartlett’s test χ^2^(351) = 13,149.39, *p* < 0.001). Table 2 presents the descriptive statistics, reliability coefficients, and correlations for the five *a priori* domains.

**Table 2:** MSUPS content domains: item allocation, descriptive statistics, internal consistency, and correlations with the study variables (*N* = 973).

Content domain	Items	*k*	*M* (SD)	*α*	*ω*	CITC	*r* with AP	*r* with GSE	*r* with self-reported daily use
D1. Compulsive use and loss of control	4, 5, 9, 12, 13, 17	6	2.91 (0.85)	0.78	0.79	0.43–0.66	0.16^***^	−0.04	0.30^***^
D2. Psychological dependence	1, 2, 6, 14, 16, 20, 25	7	2.71 (0.94)	0.89	0.89	0.57–0.77	0.18^***^	−0.07^*^	0.32^***^
D3. Functional interference	3, 11, 15, 18, 19, 22	6	2.52 (0.89)	0.82	0.83	0.41–0.70	0.18^***^	−0.13^***^	0.21^***^
D4. Impairment of social functioning	7, 8, 21, 23	4	2.22 (0.86)	0.71	0.72	0.44–0.54	0.19^***^	−0.20^***^	0.21^***^
D5. Compensatory use	10, 24, 26, 27	4	2.54 (0.87)	0.69	0.69	0.43–0.51	0.18^***^	−0.13^***^	0.25^***^
Total MSUPS score	1–27	27	2.62 (0.76)	0.94	0.94	0.42–0.75	0.21^***^	−0.12^***^	0.31^***^

AP, academic procrastination; GSE, general self-efficacy; *k*, number of items; *M*, mean; SD, standard deviation; *α*, Cronbach’s alpha; *ω*, McDonald’s omega; CITC, range of corrected item–total correlations. The five domains are content categories defined a priori for item development and are not empirically derived factors or validated subscales; they are reported here for content coverage only, and the study hypotheses were tested with the global score (see Sections 2.4.1 and 3.3). All domain scores ranged from 1 to 5. Intercorrelations among the five domains ranged from 0.60 to 0.75. Full item wording is provided in Supplementary Table S1. ^*^p < 0.05. ^***^p < 0.001.

The internal consistency of the five domains ranged from acceptable to good (*α* = 0.69–0.89; ω = 0.69–0.89), with corrected item–total correlations between 0.41 and 0.77. The lowest values were observed for compensatory use (4 items; *α* = 0.69) and impairment of social functioning (4 items; *α* = 0.71). Correlations among the five domains were high (*r* = 0.60–0.75), indicating substantial overlap between the proposed facets. Associations of the domains with academic procrastination were relatively homogeneous (*r* = 0.16–0.19), whereas associations with general self-efficacy (*r* = −0.04 to −0.20) and with daily use duration (*r* = 0.21–0.32) were more variable. Given the high intercorrelations, the domain scores were retained as content-development markers and were not interpreted as validated subscales.

### 3.3 Initial psychometric evaluation of the MSUPS

#### 3.3.1 Competing confirmatory models and the general factor

The *a priori* structure was examined on the full sample (*N* = 973) using polychoric correlations and the WLSMV estimator. The fit indices for all models are reported in Table 3. The correlated five-factor model fitted better than the one-factor model, and the second-order model showed comparable fit, with high second-order loadings (0.87–0.97) and polychoric correlations among the five factors between 0.75 and 0.93, indicating a substantial proportion of shared variance.

**Table 3:** Fit indices for the competing confirmatory factor models of the MSUPS (WLSMV estimation on polychoric correlations).

Model	Scaled χ^2^	*df*	CFI	TLI	RMSEA	SRMR
Panel A. Full sample (*N* = 973)						
One-factor	2,232.3	324	0.900	0.892	0.078	0.080
Correlated five-factor	1,783.7	314	0.923	0.914	0.069	0.070
Second-order (five domains → general factor)	1,815.8	319	0.922	0.914	0.069	0.072
Bifactor (general + five specific factors)	1,509.8	297	0.937	0.925	0.065	0.064
Panel B. Second half of the sample (*n* = 487)						
One-factor	1,348.8	324	0.886	0.877	0.081	0.086
*A priori* five-factor	1,103.0	314	0.912	0.902	0.072	0.076
Empirical three-factor (independent clusters)	1,010.0	321	0.924	0.916	0.066	0.072
Exploratory three-factor with cross-loadings (ESEM)	441.3	273	0.981	0.976	0.036	0.035

CFI, comparative fit index; TLI, Tucker–Lewis index; RMSEA, root mean square error of approximation; SRMR, standardized root mean square residual; ESEM, exploratory structural equation modeling. All models were estimated on polychoric correlations by diagonally weighted least squares with mean- and variance-adjusted test statistics (WLSMV; delta parameterization); *χ*^2^ values are scaled. Conventional criteria: CFI and TLI ≥ 0.95; RMSEA and SRMR ≤ 0.08 (Hu and Bentler, 2009). In the bifactor model, general-factor loadings ranged from 0.49 to 0.80 in the full sample (total *ω* = 0.96; *ω*ₕ = 0.92) and from 0.42 to 0.79 in the second half (*ω*ₕ = 0.91); no negative uniquenesses were obtained. Second-order loadings ranged from 0.87 to 0.97, and polychoric correlations among the five first-order factors from 0.75 to 0.93. The largest residual correlations of the bifactor model occurred between items 12 and 18 (0.25) and between items 3 and 4 (0.23). Panel B models were re-estimated in the second half of the split-sample internal evaluation described in Section 3.3.2, that evaluation provides internal cross-validation within the present sample and not independent validation of the instrument.

Of the models tested, the bifactor model showed the most favorable fit indices: χ^2^(297) = 1,509.8, CFI = 0.937, TLI = 0.925, RMSEA = 0.065, SRMR = 0.064. In this model, each item loaded simultaneously on a general factor and its domain-specific factor; general-factor loadings ranged from 0.49 to 0.80, with no negative uniquenesses. Total omega was 0.96 and hierarchical omega was ωH = 0.92, indicating that most of the reliable variance in the total score can be attributed to the general factor. The fit was therefore acceptable by the conventional RMSEA and SRMR criteria, but CFI remained below the strict 0.95 threshold; interpretation of the global score within the present sample is accordingly supported, whereas independent interpretation of the subscales is not. Inspection of the residual correlations identified the principal sources of misfit: the largest residuals occurred between items 12 and 18 (residual = 0.25) and between items 3 and 4 (residual = 0.23), pairs with appreciable semantic overlap (unintended use and reduced productivity; distraction and exceeding intended time). These results indicate item-level content redundancy and provide a concrete direction for refining the instrument.

#### 3.3.2 Split-sample evaluation of the MSUPS structure

Because the development, psychometric evaluation, and application of the instrument were carried out in the same sample, the structure was subjected to a split-sample internal evaluation by randomly splitting the sample into an exploratory half (*n* = 486) and a second half (*n* = 487). In the exploratory half, the correlation matrix was suitable for factor analysis (KMO = 0.95; Bartlett’s test χ^2^(351) = 7,136.6, *p* < 0.001). Parallel analysis indicated three dimensions rather than five: the observed eigenvalues for the first three factors were 11.38, 1.92, and 1.58, exceeding the random reference values of 1.52, 1.44, and 1.38, whereas the fourth observed eigenvalue (0.92) fell below its reference value (1.33). Principal-axis factoring with Promax rotation on three factors explained 17.1%, 14.3%, and 11.5% of the variance, respectively. The three-factor solution comprised a psychological dependence and need-for-access dimension, a loss-of-control and functional-interference dimension, and a social-consequences and avoidance-compensation dimension, with several substantial cross-loadings. Correlations among the three rotated factors were moderate to high (0.54–0.63), which supports the presence of a common component but does not at present justify the use of three independent subscale scores.

Repeating the exploratory analysis in the second half produced a very similar configuration, with Tucker congruence coefficients of 0.992, 0.992, and 0.991 for the three factors, reflecting very high between-half congruence of the loading pattern. This result indicates the stability of the loading pattern within the same recruitment context and should not be confused with external replication or measurement invariance. The internal consistency of the global score was equivalent in the two halves (*α* = 0.945 and *α* = 0.937), and the predominance of the general factor was also observed in the second half (ωH = 0.91; general-factor loadings 0.42–0.79).

In the second half, the simple-structure confirmatory models remained suboptimal (Table 3): the one-factor model, the *a priori* five-factor model, and the empirical three-factor model all fell short of the conventional CFI criterion, whereas an exploratory three-factor model allowing cross-loadings fit considerably better (CFI = 0.981, TLI = 0.976, RMSEA = 0.036, SRMR = 0.035). The difference is compatible with the view that an independent-clusters confirmatory structure is too restrictive for these items because that model estimates more free parameters; however, the result is not interpreted as definitive evidence for the superiority of an exploratory structural equation modeling solution.

Taken together, these results support use of the global score in the present sample but do not confirm the number of facets postulated *a priori*. The five-domain structure remains provisional and requires validation in independent samples.

#### 3.3.3 Measurement invariance across educational stages

Under continuous treatment of the items, metric invariance was supported, Δχ^2^(22) = 32.78, *p* = 0.065, ΔCFI = −0.001. For scalar invariance, the ΔCFI criterion was met (−0.007) but the χ^2^ difference test was significant, Δχ^2^(22) = 107.54, *p* < 0.001; therefore, scalar invariance was only partially supported.

Because treating ordinal items as continuous is inadequate, invariance was re-examined using polychoric correlations following the sequence recommended for ordered-categorical data (Wu and Estabrook, 2025). The equality of thresholds across the two groups was not supported (Wald test on 108 thresholds: W = 219.01, df = 108, *p* < 0.001), although the differences were modest in absolute magnitude (mean |Δτ| = 0.14; maximum = 0.43), with the largest deviations for items 8 and 12 (maximum |*z*| = 3.65). The equality of factor loadings was likewise not supported in the bifactor model, Δχ^2^(54) = 363.22, *p* < 0.001. Under the estimation appropriate for ordinal items, measurement invariance is therefore not established beyond the configural level, and the conclusion of metric invariance obtained under continuous treatment does not hold. These results must be interpreted within the limits of the baseline model fit. Given these findings, no comparisons of latent means were conducted between the two groups, and the between-group difference reported in Section 3.10 is interpreted strictly as a difference in the strength of an observed association between scores and not as a difference at the level of the latent construct.

### 3.4 Psychometric properties of the academic procrastination index

Because the academic procrastination measure is an unvalidated conceptual adaptation of PASS and constitutes the primary criterion of this study, its properties were examined explicitly. The six items, with full wording in Romanian and English translation, are reported in Supplementary Table S2, together with per-item descriptive statistics and corrected item–total correlations (0.55, 0.64, 0.27, 0.61, 0.67, and 0.64). A one-factor confirmatory model estimated on polychoric correlations showed unsatisfactory fit, χ^2^(9) = 196.9, RMSEA = 0.147, SRMR = 0.116, with the loading of item 3 (“reading the recommended bibliography”) markedly lower than the others (0.31 versus 0.63–0.83). Removing this item improved both internal consistency (*α* = 0.83 versus 0.80) and model fit, χ^2^(5) = 55.4, RMSEA = 0.102, SRMR = 0.076, without altering the substantive conclusions: the association with problematic smartphone use remained equivalent (*r* = 0.19, *p* < 0.001, versus *r* = 0.21 for the six-item score). Therefore, the scale cannot be regarded as a validated unidimensional instrument; it functions as a composite index of delay frequency with heterogeneous content coverage; the sensitivity analysis excluding the weakest item addresses this only partly and does not substitute for independent psychometric validation. This limitation is discussed in Section 4.8.

### 3.5 Assumption checks

Assumption checks indicated no problems with the distribution of residuals, independence of observations, multicollinearity, or influential observations (Durbin–Watson = 1.99; VIF ≤ 1.11; maximum Cook’s distance = 0.031). The Breusch–Pagan test indicated heteroscedasticity (LM = 53.46, *p* < 0.001); HC3 heteroscedasticity-robust standard errors were therefore used in all regression models.

### 3.6 Correlations

The correlation matrix is presented in Table 4. Problematic smartphone use was positively associated with academic procrastination and daily use duration and negatively with general self-efficacy. Daily use duration and general self-efficacy were not significantly associated with academic procrastination. Age was not associated with problematic smartphone use or procrastination but showed a weak positive association with general self-efficacy.

**Table 4:** Pearson correlations among the study variables with 95% confidence intervals (*N* = 973; *n* = 971 for age).

Variable	1	2	3	4	5
1. Problematic smartphone use	—				
2. General self-efficacy	−0.12^***^ [−0.19, −0.06]	—			
3. Academic procrastination	0.21^***^ [0.15, 0.27]	0.06 [−0.00, 0.12]	—		
4. Self-reported daily smartphone use (hours)	0.31^***^ [0.25, 0.37]	0.01 [−0.05, 0.08]	0.04 [−0.02, 0.10]	—	
5. Age (categories)	−0.02 [−0.08, 0.04]	0.11^***^ [0.04, 0.17]	0.02 [−0.04, 0.09]	−0.07^*^ [−0.13, −0.01]	—

Values in brackets are 95% confidence intervals obtained by Fisher’s *z* transformation. Age was collected in categories and recoded to representative values correlations involving age are based on *n* = 971. ^*^p < 0.05. ^***^p < 0.001.

### 3.7 Problematic smartphone use and academic procrastination (H1)

Problematic smartphone use was positively associated with academic procrastination, *r*(971) = 0.21, 95% CI [0.15, 0.27], *p* < 0.001. In a linear regression model, problematic use accounted for a small proportion of the variance in academic procrastination, *b* = 0.26, SE = 0.04, 95% CI [0.18, 0.33], *β* = 0.21, *R*^2^ = 0.04, *F*(1, 971) = 43.97, *p* < 0.001, f^2^ = 0.05. This result was maintained with HC3 robust standard errors and Spearman’s coefficient (*ρ* = 0.20, *p* < 0.001). H1 was therefore supported, with an effect of small magnitude.

### 3.8 Problematic use characteristics versus self-reported daily use duration (H2)

Self-reported daily smartphone use duration was not significantly associated with academic procrastination (*r* (971) = 0.04, 95% CI [−0.02, 0.10], *p* = 0.192), although it was associated with problematic use (*r* = 0.31, *p* < 0.001). The association between problematic use and procrastination (*r* = 0.21) was significantly stronger than the association between self-reported use duration and academic procrastination (Williams test *t*(970) = 4.50, *p* < 0.001, difference = 0.17).

Given that duration was measured with a single item and that the highest category was open-ended, robustness was assessed through additional analyses. The Spearman correlation computed on the original ordinal categories was statistically significant but negligible in magnitude (*ρ* = 0.06, *p* = 0.044); at *N* = 973, a coefficient of this size reaches significance without practical relevance, and the substantive interpretation therefore rests on the convergence of the full set of sensitivity analyses rather than on any single significance test. Differences across the six categories were not significant (Kruskal–Wallis H (5) = 10.94, *p* = 0.053, ε ^2^ = 0.006). In the subsample excluding the open-ended upper category (*n* = 623), self-reported use duration was not associated with procrastination (*r* = −0.05, *p* = 0.242), while problematic use retained its association, although at reduced magnitude (*r* = 0.15, *p* < 0.001). Results also remained similar after correction for attenuation. Estimates obtained under the alternative operationalizations are presented comparatively in Figure 2.

**Figure 2:**
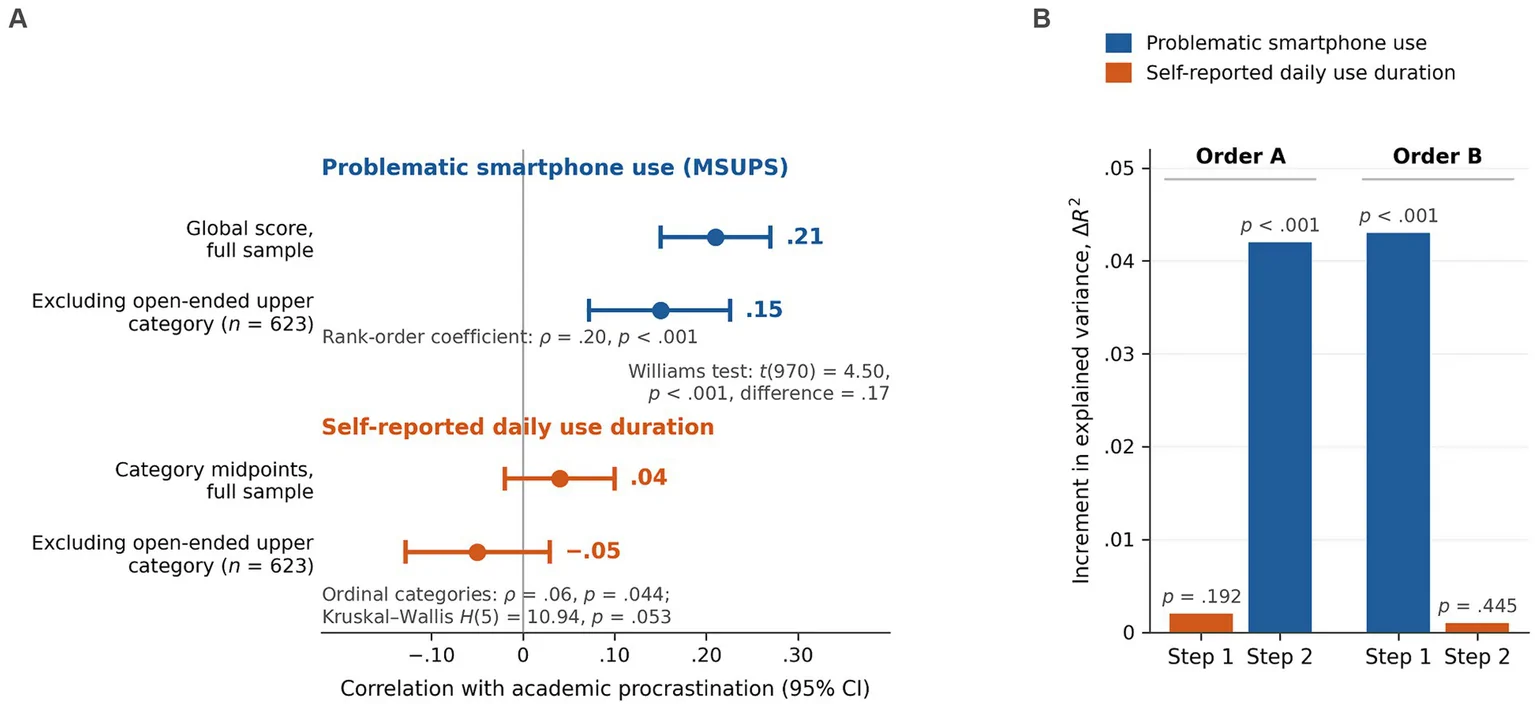
Problematic smartphone use and self-reported daily use duration as predictors of academic procrastination (*N* = 973). **(A)** Pearson correlations with academic procrastination and 95% confidence intervals obtained by Fisher’s *z* transformation, under alternative operationalizations of the two predictors; the subsample analyses exclude participants in the open-ended upper duration category (*n* = 623). **(B)** Increment in explained variance in the hierarchical regressions under the two predictor entry orders; *p* values refer to the *F* test of Δ*R*^2^.

Hierarchical regression confirmed the difference between the two predictors (Table 5). After controlling for use duration, problematic use explained additional variance in academic procrastination, Δ*R*^2^ = 0.042, *F*(1, 970) = 42.76, *p* < 0.001. Conversely, use duration explained no additional variance once problematic use was entered, Δ*R*^2^ = 0.001, *F*(1, 970) = 0.58, *p* = 0.445; the corresponding HC3 Wald test for the same coefficient led to the same conclusion, *p* = 0.464. In the simultaneous model, problematic use remained the only significant predictor (*β* = 0.22, *p* < 0.001), whereas use duration was not significant (*β* = −0.03, *p* = 0.464). H2 was supported.

**Table 5:** Hierarchical regression analyses predicting academic procrastination under the two alternative predictor entry orders (*N* = 973).

Model and step	*R* ^2^	Δ*R*^2^	*F* for Δ*R*^2^	*p*	*b*	*β*
Order A: self-reported duration entered first						
Step 1: self-reported daily smartphone use	0.002	0.002	1.71	0.192	—	—
Step 2: + problematic smartphone use	0.044	0.042	42.76	< 0.001	0.268	0.216
Order B: problematic use entered first						
Step 1: problematic smartphone use	0.043	0.043	43.97	< 0.001	0.258	0.208
Step 2: + self-reported daily smartphone use	0.044	0.001	0.58	0.445	−0.020	−0.025

*b*, unstandardized regression coefficient; *β*, standardized regression coefficient; Δ*R*^2^, change in explained variance. Degrees of freedom were (1, 971) at Step 1 and (1, 970) at Step 2. The *F* tests refer to the change in *R*^2^ and are based on ordinary least squares. All coefficients were re-estimated with HC3 heteroscedasticity-robust standard errors, which did not alter any conclusion; for daily smartphone use at Step 2 of Order B the HC3 Wald test gave *p* = 0.464. The difference between the two dependent correlations with academic procrastination was significant (Williams test, *t*(970) = 4.50, *p* < 0.001, difference = 0.17).

### 3.9 General self-efficacy as an intervening variable (H3)

The results of the indirect-effect model are presented in Table 6. The term “indirect effect” refers throughout to a statistical decomposition of an observed association and not to mediation, which this design cannot test. Problematic smartphone use was associated with lower general self-efficacy (*a* = −0.09, SE = 0.03, *p* = 0.001). After controlling for problematic use, general self-efficacy showed a weak positive association with academic procrastination (*b* = 0.14, SE = 0.06, *p* = 0.014) in the direction opposite to the *a priori* hypothesis.

**Table 6:** Indirect-effect model: problematic smartphone use → general self-efficacy → academic procrastination (*N* = 973).

Path	*b*	SE (HC3)	*t*	*p*	95% CI
*a*: Problematic smartphone use → General self-efficacy	−0.092	0.029	−3.20	0.001	[−0.148, −0.036]
*b*: General self-efficacy → Academic procrastination (controlling for PSU)	0.145	0.059	2.46	0.014	[0.030, 0.260]
*c*: Total effect	0.258	0.044	5.90	<0.001	[0.173, 0.344]
*c*′: Direct effect	0.272	0.044	6.18	<0.001	[0.185, 0.358]
*ab*: Indirect effect (percentile bootstrap, 5,000 resamples)	−0.013	—	—	—	[−0.028, −0.002]

PSU, problematic smartphone use; *b*, unstandardized coefficient; SE, standard error; CI, confidence interval. HC3 heteroscedasticity-robust standard errors were used for paths a, b, c, and c′; the confidence interval for the indirect effect was obtained by percentile bootstrap with 5,000 resamples. Standardized coefficients were *β* = 0.208 for the total effect and *β* = 0.219 for the direct effect; the completely standardized indirect effect was −0.01. The model is a statistical decomposition of association and does not establish temporal mediation. In a sensitivity analysis excluding respondents with invariant responding on the general self-efficacy scale (*n* = 863), path b was no longer significant (*b* = 0.06, *p* = 0.348).

The indirect effect was small, *ab* = −0.013, 95% bootstrap CI [−0.028, −0.002], and the completely standardized indirect effect was −0.01. The total effect (*c* = 0.258, *β* = 0.208) and direct effect (*c*′ = 0.272, *β* = 0.219) were similar. In addition, in a sensitivity analysis excluding participants with invariant responses on the self-efficacy scale, the association between self-efficacy and procrastination did not hold (*b* = 0.06, *p* = 0.348).

Therefore, although the indirect effect differed statistically from zero in the primary analysis, the direction of path b, the small magnitude of the effect, and the outcome of the sensitivity analysis did not support the hypothesized indirect association. H3 was not supported.

### 3.10 Exploratory moderation by educational stage (RQ1)

The exploratory analysis indicated a significant interaction between problematic smartphone use and educational stage (*b* = 0.220, HC3 SE = 0.092, *t* = 2.41, *p* = 0.016, Δ*R*^2^ = 0.007) (Table 7). Simple slope analysis (Figure 3) showed that the association with academic procrastination was significant in both groups but stronger in the secondary education group (*b* = 0.402, HC3 SE = 0.074, *p* < 0.001) than in the higher education group (*b* = 0.182, HC3 SE = 0.054, *p* < 0.001). The correlation was likewise higher in the secondary-education group (*r* = 0.31, 95% CI [0.21, 0.41], *R*^2^ = 0.098) than in the higher-education group (*r* = 0.15, 95% CI [0.07, 0.22], *R*^2^ = 0.022), and the difference between coefficients was significant (Fisher’s *z* = 2.56, *p* = 0.011).

**Table 7:** Exploratory moderation by educational stage: regression coefficients and simple slopes (*N* = 973).

Predictor/simple slope	*b*	SE (HC3)	*t*	*p*	95% CI
Problematic smartphone use (mean-centered)	0.182	0.054	3.36	< 0.001	[0.076, 0.288]
Educational stage (1 = secondary education)	0.000	0.063	0.00	0.997	[−0.123, 0.123]
Problematic smartphone use × Educational stage	0.220	0.092	2.41	0.016	[0.041, 0.399]
Simple slope: higher-education students	0.182	0.054	3.36	< 0.001	[0.076, 0.288]
Simple slope: secondary-education students	0.402	0.074	5.46	< 0.001	[0.258, 0.547]

CI, confidence interval. The continuous predictor was mean-centered; educational stage was coded 0 = higher education and 1 = secondary education. HC3 heteroscedasticity-robust standard errors were used. *R*^2^ for the full interaction model = 0.050; the interaction term accounted for Δ*R*^2^ = 0.007. The association was significant in both groups but stronger in secondary education (*r* = 0.31, 95% CI [0.21, 0.41], *R*^2^ = 0.098) than in higher education (*r* = 0.15, 95% CI [0.07, 0.22], *R*^2^ = 0.022); the difference between the two coefficients was significant (Fisher’s *z* = 2.56, *p* = 0.011). Because measurement invariance was not established beyond the configural level under estimation appropriate to ordinal items, this result is interpreted as a difference in the strength of an observed association and not as a difference at the latent level. The analysis is exploratory.

**Figure 3:**
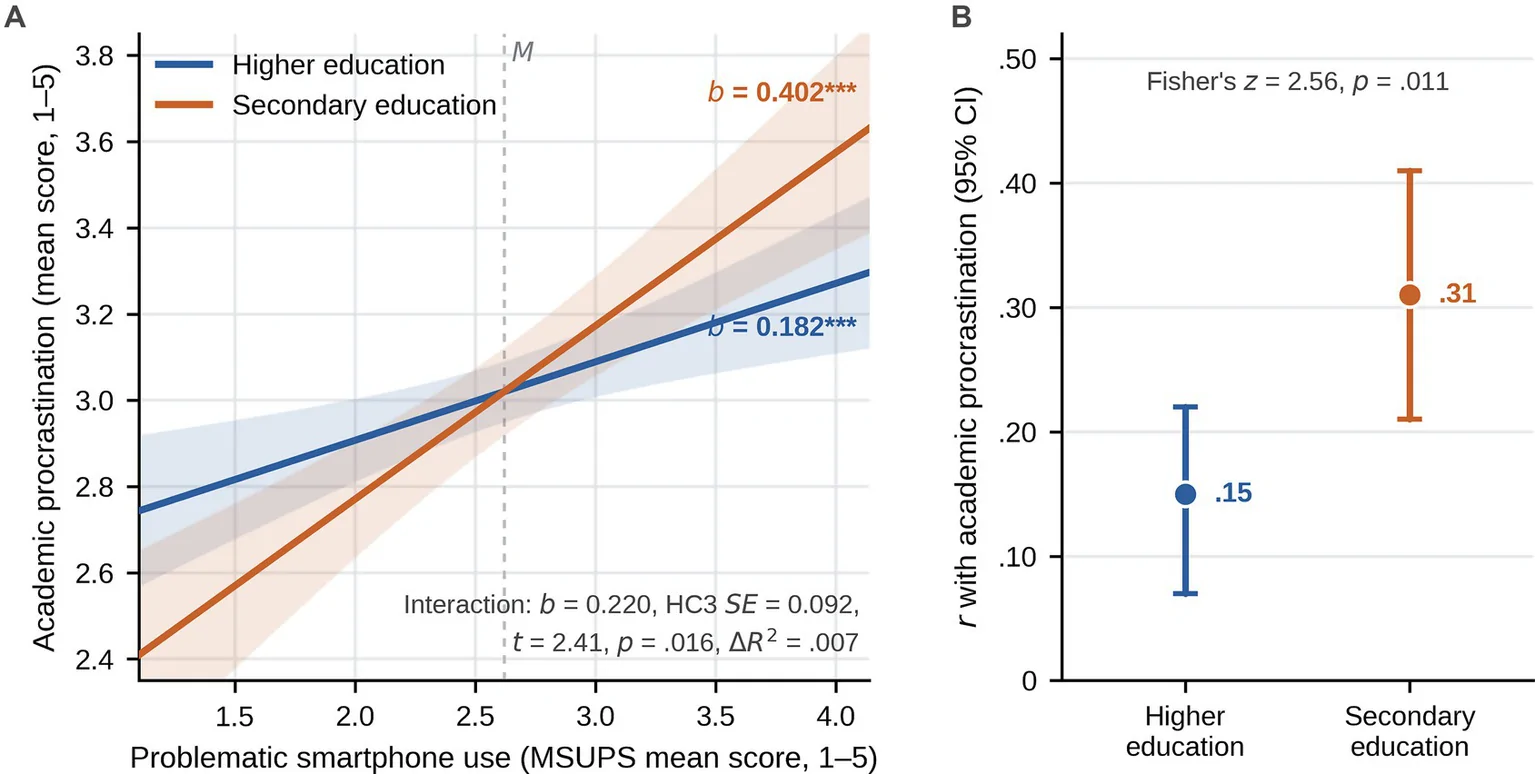
Association between problematic smartphone use and academic procrastination by educational stage (*N* = 973). **(A)** Simple slopes from the moderation model with heteroscedasticity-robust (HC3) standard errors; the predictor was mean-centered in the model and is displayed on its original metric, the dashed line marks the sample mean, and shaded areas are 95% confidence bands. **(B)** Correlations within each group with 95% confidence intervals and the test of the difference between them. The secondary-education group consists of adult students aged 18 years or older in their final high-school years; because measurement invariance was not established beyond the configural level, the difference concerns observed scores only. ^***^p < 0.001.

Because the moderator is a derived variable, the interaction was submitted to a series of sensitivity analyses, varying the coding decisions (Section 2.6). The interaction remained significant in all variants: reclassifying the 13 multiply marked cases as higher education (*b* = 0.205, *p* = 0.027), excluding them (*b* = 0.217, *p* = 0.020), excluding the 14 participants with indications of post-secondary non-tertiary education (*b* = 0.215, *p* = 0.020), and the independent recoding based on the institution field (*b* = 0.201, *p* = 0.042, *n* = 822). The exploratory conclusion, therefore, does not depend on the coding of ambiguous cases. Re-estimated separately by gender, the interaction was significant in the female subsample (*b* = 0.267, *p* = 0.022, *n* = 667) but not in the male subsample (*b* = 0.096, *p* = 0.571, *n* = 296), where statistical power is substantially lower; this difference does not permit conclusions about moderation by gender. In a further analysis, the interaction with age category was not significant (*b* = −0.029, SE = 0.019, *p* = 0.127), this variable being available only in categorical form.

These exploratory results therefore indicate a difference in the strength of the observed association between the two educational stages rather than a developmental difference: given the minimum age criterion of 18 years, the comparison contrasts adult students in their final high school years with university students, not adolescents with adults (Sections 2.3 and 4). Moreover, because measurement invariance of the MSUPS was not established beyond the configural level under ordinal estimation (Section 3.3.3), the difference is interpreted at the level of observed scores and not as a difference at the latent level.

### 3.11 Common method variance

Harman’s single-factor test, applied to the 43 items of the main scales, showed that the first unrotated factor accounted for 26.2% of the variance in the data. This does not indicate a single dominant factor, but given the limitations of Harman’s test, it does not exclude the possibility of common method variance (Podsakoff et al., 2003).

### 3.12 Sensitivity analyses

Conclusions were maintained when the five-item procrastination score was used, obtained by removing the item with the low corrected item–total correlation, and when the analysis was repeated on the full sample before screening (*r* = 0.21). When participants with invariant responses on the self-efficacy scale were excluded (*n* = 863), the main association was maintained (*r* = 0.18), but path b in the indirect effect model fell to 0.06 and became non-significant (*p* = 0.348). The primary association between problematic smartphone use and academic procrastination was robust across all these checks, although such checks cannot exclude inflation by common-method variance; that robustness does not extend to the secondary findings involving general self-efficacy, which were sensitive to the exclusion of invariant responders. The sensitivity of path b is compatible with the possible influence of response style on the positive association between self-efficacy and procrastination, given the absence of reverse-worded items across all instruments used, although it does not permit acquiescence or careless responding to be identified with certainty.

Four further checks were conducted. The main association was invariant across gender (female *r* = 0.189, *n* = 667; male *r* = 0.216, *n* = 296; Fisher’s *z* = −0.39, *p* = 0.698). Removing item 8 (“People close to me have expressed concern about the time I spend on my smartphone”), the only item worded from the perspective of others’ perceptions, changed neither reliability (*α* = 0.939 versus 0.941) nor the main association (*r* = 0.207 versus 0.208). Participants with invariant responding on the self-efficacy scale (*n* = 110) did not differ from the rest of the sample in problematic use (*d* = −0.21, *p* = 0.078) or procrastination (*d* = −0.09, *p* = 0.481) but reported substantially higher self-efficacy (*M* = 3.34 versus 3.05; *d* = 0.50, *p* = 0.001), a pattern consistent with systematic endorsement of the maximum value on that scale rather than with generalized random responding. Finally, the educational-stage moderation was maintained when the five-item procrastination score was used.

To exclude the possibility that the main association was produced artificially by content overlap between the MSUPS and the procrastination criterion, the problematic use score was recomputed without the four items referring directly to distraction, task avoidance, productivity, and academic interference (items 3, 10, 18, and 19). The restricted 23-item score (*α* = 0.93) correlated *r* = 0.99 with the full score and preserved the entire pattern of results: the association with academic procrastination remained essentially unchanged (*r* = 0.21, 95% CI [0.15, 0.27], *p* < 0.001), the difference from self-reported use duration remained significant (Williams test *t*(970) = 4.69, *p* < 0.001), over self-reported duration was maintained (Δ*R*^2^ = 0.044), and the interaction with educational stage remained of comparable magnitude (*b* = 0.227; *r* = 0.32 in the secondary-education group and *r* = 0.15 in the higher-education group). This sensitivity analysis therefore provides little support for the explanation that the observed association is driven solely by content overlap at the item level. However, it does not exclude conceptual overlap between the constructs assessed and does not establish discriminant validity between them.

### 3.13 Exploratory analysis of self-reported reasons for procrastination

Eight reasons for procrastination were analyzed exploratorily and separately from hypothesis testing. Parallel analysis indicated two dimensions, and principal-axis factoring with Promax rotation identified a task-aversion and self-regulation-difficulty factor (six items; *α* = 0.82) and a fear-of-failure and perfectionism factor (two items), which were weakly correlated (Φ = 0.22). The first dimension was positively associated with problematic smartphone use (*r* = 0.50, 95% CI [0.45, 0.54]) and negatively with general self-efficacy (*r* = −0.12, 95% CI [−0.19, −0.06]), whereas the second showed a weaker association with problematic use (*r* = 0.26, 95% CI [0.20, 0.32]) and a positive association with self-efficacy (*r* = 0.12, *p* < 0.001). Entered together with problematic use in the prediction of academic procrastination, the two dimensions explained additional variance, Δ*R*^2^ = 0.038, *F*(2, 969) = 19.91, *p* < 0.001. Perfectionism was the most frequently endorsed reason but showed the weakest integration with the remaining items (corrected item–total correlation = 0.07). Full descriptive statistics and correlations are reported in Supplementary Table S3. Because these analyses were exploratory, significance thresholds were not adjusted for multiple comparisons, and the results did not alter the central conclusions. The *post hoc* analysis of age at first mobile phone ownership and current daily smartphone use is reported in Supplementary Table S6.

## 4 Discussion

### 4.1 Main findings

This study examined whether academic procrastination is associated more closely with problematic smartphone use than with the self-reported duration of that use, tested general self-efficacy as a candidate intervening variable, and provided an initial psychometric evaluation of the MSUPS. H1 and H2 were supported and H3 was not. Problematic smartphone use was positively associated with academic procrastination, whereas self-reported daily use duration was not, and self-reported duration explained no additional variance once problematic use was entered. General self-efficacy did not account for the association in the hypothesized direction. An exploratory analysis indicated a stronger association among participants in secondary than in higher education. The psychometric analyses showed high reliability of the global score and a dominant general factor, while leaving the facet structure unresolved. Given the cross-sectional design, all results are interpreted as associations and permit no causal inference. The psychometric evidence for the MSUPS remains preliminary and confined to the present sample, and the procrastination criterion is a conceptually adapted index whose proposed one-factor structure was not supported. The substantive findings therefore require replication with independently validated measures of both constructs.

### 4.2 Problematic use characteristics versus self-reported duration

The central result concerns the distinct standing of problematic smartphone use relative to self-reported time spent on the device. Although the two variables were moderately correlated (*r* = 0.31), only problematic use was associated with academic procrastination and explained additional variance beyond self-reported duration; the difference between the two associations was formally significant. This pattern is consistent with the literature treating time of use, considered in isolation, as a limited and non-specific indicator (Billieux et al., 2015; Panova and Carbonell, 2018).

Three qualifications bound this interpretation. First, the result did not depend on how duration was operationalized: the same conclusion followed under the original ordinal categories, non-parametric testing, exclusion of the open-ended upper category, and correction for attenuation. Second, the non-parametric test on the ordinal categories was nevertheless statistically significant, albeit negligible in magnitude (*ρ* = 0.06), so the finding is better described as a negligible rather than an absent association. Third, and most importantly, the comparison is asymmetrical in measurement quality: a 27-item scale with *α* = 0.941 was compared with a single self-report item whose highest category was open-ended and contained more than a third of the sample. Correction for attenuation and the alternative operationalizations mitigate but do not eliminate this asymmetry. It is also notable that in the subsample excluding the open-ended upper category the association of problematic use with procrastination itself fell from *r* = 0.21 to *r* = 0.15, which suggests that range restriction on the duration variable affects both predictors rather than only one. The finding therefore indicates a stronger association for problematic characteristics of use than for the single-item self-report of duration used here. It does not establish that problematic-use measures are generally superior to accurately or objectively measured smartphone exposure, nor that objectively recorded screen time is irrelevant to academic delay.

Two further measurement constraints bear on the central association. First, academic procrastination was assessed with an unvalidated conceptual adaptation whose one-factor model showed unsatisfactory fit, largely because of the weak loading of item 3 (“reading the recommended bibliography”), and the resulting score functions as a composite index of delay frequency. The central association therefore links a newly developed predictor to an adapted criterion, and the statistical precision of the estimates should not be read as evidence for the construct validity of either measure. Conclusions about the relationship between smartphone use and academic procrastination are therefore conditional on this outcome measure. Second, the association was essentially unchanged when the four MSUPS items closest in content to academic delay were removed (Section 3.12), but item removal addresses overlap at the item level only. Loss of control over an activity that displaces academic work remains conceptually close to academic delay, so discriminant validity between the two constructs has not been established, and replication with established independent measures would be needed to settle this.

### 4.3 Interpretation in terms of self-regulation

The pattern is compatible with a self-regulation reading. What the MSUPS captures - unintended use, difficulty reducing use, low resistance to checking urges, and interference with sleep and responsibilities - describes failures of monitoring and adjustment rather than exposure as such, and these are precisely the processes whose breakdown defines procrastination within self-regulated learning models (Steel, 2007; Wolters, 2003; Zimmerman, 2002). The exploratory analysis of self-reported reasons is consistent with this reading: problematic use was associated roughly twice as strongly with the task-aversion and self-regulation-difficulty dimension as with the fear-of-failure and perfectionism dimension, and only the former was negatively associated with self-efficacy. This pattern is consistent with recent evidence that self-regulatory processes do not uniformly offset motivational deficits: Roman et al. (2026) found that amotivation remained directly associated with academic engagement regardless of perceived performance control, and that the contributions of forethought and self-reflection were conditional rather than compensatory.

Two limits on this interpretation should be stated plainly. The data are cross-sectional, so they are equally compatible with the reverse ordering, in which students who habitually delay academic work turn to the smartphone as an available alternative, and with a common third factor such as trait impulsiveness. Nothing in this design distinguishes these accounts. Furthermore, the effect was small: problematic use accounted for approximately 4% of the variance in academic procrastination (*R*^2^ = 0.04; f^2^ = 0.05). Procrastination is multiply determined, and problematic smartphone use is one of the factors associated with it rather than a major determinant. This calibration limits the risk of over-interpreting a statistically significant result obtained in a large sample.

### 4.4 General self-efficacy did not account for the association (H3)

Contrary to H3, general self-efficacy did not explain the association between problematic smartphone use and academic procrastination in the direction hypothesized. Problematic use was associated with lower self-efficacy, in line with theory (Bandura, 1997), but path b was positive: after controlling for problematic use, higher self-efficacy was associated with slightly more procrastination. The resulting indirect effect was negative and of negligible magnitude (completely standardized *ab* = −0.01), a pattern compatible with statistical suppression, and it did not survive exclusion of respondents with invariant responding on the self-efficacy scale. The decomposition is statistical; with cross-sectional data it cannot establish temporal order among the three variables.

Rather than developing a *post hoc* account of this reversal, we note that it is most plausibly a measurement artifact and a construct-specificity problem. The sensitivity analysis is compatible with a possible influence of response style - none of the instruments contained reverse-worded items - but it does not permit acquiescence or careless responding to be identified with certainty. The two sets of findings should therefore be weighted differently: the primary association remained significant in every sensitivity analysis conducted (*r* = 0.15 to 0.22), whereas the self-efficacy path did not, and the latter is reported as an exploratory observation rather than as a robust result. Independently of this, the zero-order association between general self-efficacy and academic procrastination was weak and non-significant (*r* = 0.06), whereas studies that have identified intervening effects have generally assessed domain-specific forms of self-efficacy (Li et al., 2020; Liu et al., 2022; Zhao et al., 2025). Self-efficacy for self-regulated learning is conceptually closer to the initiation and maintenance of academic tasks than generalized self-efficacy is Klassen et al. (2008) and Schunk and DiBenedetto (2020). The appropriate conclusion is that H3 was not supported and that the unexpected indirect effect is an exploratory observation requiring replication, not evidence of a suppression mechanism.

### 4.5 Exploratory difference by educational stage (RQ1)

In the exploratory analysis, the association between problematic smartphone use and academic procrastination was stronger in the secondary-education group (*R*^2^ = 0.098) than in the higher-education group (*R*^2^ = 0.022), and the interaction was robust to four variations in the coding of the derived moderator. The interaction nevertheless explained only Δ*R*^2^ = 0.007, and the association was significant in both groups, so what is at issue is a difference in the strength of an association and not the presence versus absence of an association.

Three constraints must accompany this result. First, under estimation appropriate to ordinal items, measurement invariance of the MSUPS across the two groups was not established beyond the configural level. The observed difference cannot therefore be attributed with confidence to a difference at the construct level; it may partly reflect differential item functioning between the groups. The interpretation is accordingly restricted to the strength of the association between observed scores. Second, given the minimum age criterion of 18 years, the comparison contrasts adult students in their final high-school years with university students, not adolescents with adults, and cannot be generalized to high-school students as a population or to adolescents. Third, educational stage integrates differences in age and in institutional context; the analysis based on age categories reported in Section 3.10 showed no significant interaction, but the categorical measurement of age limits what that analysis can establish. Educational stage and age cannot be fully disentangled in this design. The result is exploratory and requires confirmation in independent samples before any developmental interpretation is entertained. Accordingly, no practical or developmental implications are drawn from this subgroup comparison.

### 4.6 Psychometric standing of the MSUPS

The psychometric analyses were conducted to establish whether the total score could reasonably carry the main analyses, not to validate a new instrument, and they should be read in that limited sense. The total score was highly reliable and, under categorical estimation appropriate to ordinal items, the bifactor model showed acceptable fit on RMSEA and SRMR with a dominant general factor (ωH = 0.92), which was also observed in the second half of the sample (ωH = 0.91). These findings support the use of the total score in the present study, which is the use to which the instrument was put; they do not constitute validation of the instrument beyond this sample.

At the same time, four results constrain any stronger claim. CFI for the bifactor model remained below the strict 0.95 threshold. Parallel analysis in the exploratory half recovered three dimensions rather than the five postulated *a priori*, and although the three-factor loading pattern was highly congruent across halves (Tucker coefficients ≥ 0.991), congruence within a single recruitment is not external replication. Simple-structure confirmatory models fitted less well than an exploratory model permitting cross-loadings, indicating that an independent-clusters structure is too restrictive for these items. Residual correlations identified specific item pairs with content redundancy, which gives a concrete direction for revision. The five-domain structure must therefore be treated as provisional; the five domains were useful for content coverage but are not validated subscales, and neither a five-factor nor a three-factor subscale solution is recommended for use at this stage.

Two further points bear on the interpretation of the whole study. The instrument was developed, evaluated, and applied within the same sample. The split-sample internal evaluation attenuates but does not remove this circularity, because both halves come from the same convenience sample and the same collection context. And no evidence is yet available on convergent, discriminant, or predictive validity, or on test–retest stability. The MSUPS is at a preliminary stage. The present article should therefore not be read as a validation study, and the substantive findings require replication with independently validated measures of problematic smartphone use in independent samples.

### 4.7 Strengths

The study has several methodological strengths. The sample was large (*N* = 973) and drawn from a cultural context under-represented in this literature, and the screening rule and exclusion criteria are reported transparently. Problematic use and use duration were measured in the same sample, which is what makes the central comparison possible, and the difference between the two associations was tested formally rather than inferred from the significance of one coefficient and the non-significance of the other. Hierarchical regressions were run in both predictor entry orders. The MSUPS structure was examined through four competing models and re-examined in a split-sample internal evaluation, which provides internal cross-validation but not independent validation. Ordinal measurement invariance was tested using the sequence appropriate to categorical items, and its failure is reported rather than omitted. Content overlap between predictor and criterion was tested directly by item removal. Robust standard errors, bootstrap intervals for the indirect effect, reported effect sizes, an unsupported hypothesis reported as unsupported, and explicit labeling of exploratory analyses all contribute to the transparency of the report.

### 4.8 Limitations

Several limitations qualify the conclusions, and they group into four sets.

The first concerns design. The study is cross-sectional and cannot establish temporal order or causality (Maxwell and Cole, 2007); the indirect-effect model is a statistical decomposition and does not establish temporal mediation. The study was not preregistered, and although H1–H3 were formulated *a priori*, this cannot be independently verified. RQ1 and the analyses of procrastination reasons were exploratory and require confirmation.

The second concerns measurement. All variables were self-reported at a single occasion, which raises the possibility of common method variance; Harman’s test did not indicate a single dominant factor, but that test is weak. All items across all instruments were worded in the same direction, and the sensitivity analysis on invariant responders is compatible with a response-style influence on at least one path in the model. Daily use duration was estimated with a single item with an open-ended upper, category that contained 36.0% of the sample and objective logging would provide more precise estimates. Age was collected in categories, which limits the separate examination of its contribution. The academic procrastination measure is an unvalidated conceptual adaptation that did not meet fit criteria for a one-factor model and is best regarded as a composite index of delay frequency, and the sensitivity analysis excluding the weakest item does not replace independent validation of that measure.

The third concerns the instrument on which the study depends. The MSUPS was developed and evaluated in the same sample in which the hypotheses were tested; its facet structure was not confirmed; measurement invariance across educational stages was not established beyond the configural level, which further limits between-group comparisons; and each half of approximately 486 participants provides a reasonable but not generous basis for complex ordinal models with cross-loadings. Content overlap with the criterion was addressed at the item level but cannot be excluded at the construct level, since loss of control over an activity that displaces academic work is conceptually adjacent to academic delay; discriminant validity between the two constructs has therefore not been established.

The fourth concerns generalizability. The sample was a convenience sample, unevenly distributed by gender (68.6% female), recruited within one national context, with higher-education participants from a single university and secondary-education participants from high schools in one county. The minimum age criterion of 18 years makes the secondary-education subsample atypical for its population. Generalization beyond this sample, and in particular to adolescents or to high-school students as a whole, is not warranted.

### 4.9 Directions for future research

Three priorities follow. First, future studies should measure duration, context, and loss of control over smartphone use separately, and should include objective use logs (e.g., device-recorded screen time) alongside self-report; the asymmetry in measurement quality identified here is the main threat to the central comparison and is straightforward to remove. Second, mechanism testing should use domain-specific measures - self-efficacy for self-regulated learning rather than generalized self-efficacy (Schunk and DiBenedetto, 2020; Schunk and Ertmer, 2000) - and longitudinal designs capable of addressing temporal order. Including reverse-worded items, or a marker variable, would allow response style to be separated from substance. Third, the MSUPS requires validation in independent samples, including refinement of the redundant item pairs identified here, examination of convergent and discriminant validity against established measures, test–retest assessment, and clarification of its dimensional structure, for which exploratory structural equation modeling is a suitable approach. The same applies to the procrastination criterion, which should be replaced by an independently validated measure of academic procrastination or subjected to equivalent psychometric evaluation.

Two implications may orient research on interventions, without themselves being demonstrated by the present study. Interventions might target the self-regulation of digital use - goal setting, self-monitoring, notification management, and the organization of the study context - rather than the reduction of use time alone. The exploratory difference associated with educational stage would, if confirmed independently, justify examining stage-adapted approaches in future research; the present data do not warrant stage-specific recommendations for practice. The present results do not demonstrate the effectiveness of any intervention and do not justify clinical recommendations or diagnostic thresholds for the MSUPS. Because problematic use accounted for approximately 4% of the variance in academic procrastination, smartphone-focused interventions would at most constitute one component of broader intervention approaches rather than a primary lever.

## 5 Conclusion

In this cross-sectional convenience sample of adults enrolled in secondary or higher education in Romania, academic procrastination was associated more consistently with problematic characteristics of smartphone use than with self-reported daily use duration, assessed with a single item; this comparison does not extend to objectively recorded exposure. The effect was small (approximately 4% of the variance in academic procrastination), and general self-efficacy did not account for the association in the hypothesized direction. The exploratory difference between educational stages, which accounted for a small proportion of variance (Δ*R*^2^ = 0.007), is interpreted strictly at the level of observed scores, because measurement invariance was not supported beyond the configural level under estimation appropriate to ordinal items, and because the secondary-education subsample consists of adult students in their final high-school years. The psychometric analyses support only provisional use of the MSUPS total score within this study; the instrument is not presented as validated, and the findings require replication with independently validated measures of problematic smartphone use and of academic procrastination, and with objective indicators of use. Accordingly, results based on the MSUPS global score should be regarded as preliminary until they are replicated in independent samples with independently validated instruments. The findings are consistent with a self-regulation interpretation, but the cross-sectional design permits no conclusions about temporal order or causality, and longitudinal and independently replicated research is required before such conclusions can be drawn.

## Data Availability

The original contributions presented in the study are included in the article/Supplementary material, further inquiries can be directed to the corresponding author.
