# Supplementary material for: Problematic smartphone use is more strongly associated with academic procrastination than self-reported daily use duration
Source: Front Psychol. 2026 Oct 28;17:1963771. doi: 10.3389/fpsyg.2026.1963771 (PMC13752132; doi:10.3389/fpsyg.2026.1963771)
Supplement: Supplementary file 1 [file Supplementary_file_1.DOCX]

**Supplementary Material**

This Supplementary Material presents the instruments administered in the present study and additional descriptive information. The 27-item Multidimensional Smartphone Use Problem Scale (MSUPS) was developed by the authors for this study; the item wordings were composed for the present instrument and are not a translation or adaptation of an existing scale. The six-item academic procrastination index and the eight items on reasons for procrastination were adapted conceptually from the Procrastination Assessment Scale for Students (Solomon and Rothblum, 1984) and are not a translation of that scale. The Romanian version was the one administered, through Google Forms, anonymously and in accordance with Regulation (EU) 2016/679 (GDPR); the English version is provided for international readers and was not administered to participants. The sample comprised adults enrolled in secondary (n = 342) or higher education (n = 631) in Romania (N = 973).

**Supplementary Table S1.** MSUPS items in Romanian and English, with the a priori allocation to content domains.

| **No.** | **Domain** | **Item (Romanian)** | **Item (English)** |
| --- | --- | --- | --- |
| 1 | D2 | Simt nevoia să-mi folosesc telefonul mobil din ce în ce mai des. | I feel the need to use my mobile phone more and more often. |
| 2 | D2 | Mă simt neliniștit(ă) atunci când nu am acces la telefonul mobil. | I feel restless when I do not have access to my mobile phone. |
| 3 | D3 | Telefonul mobil îmi distrage atenția de la alte activități importante. | My mobile phone distracts me from other important activities. |
| 4 | D1 | Petrec mai mult timp pe telefon decât intenționez. | I spend more time on my phone than I intend to. |
| 5 | D1 | Îmi verific telefonul chiar dacă nu am nicio notificare. | I check my phone even when I have not received any notifications. |
| 6 | D2 | Mă simt iritat(ă) dacă nu pot folosi telefonul mobil. | I feel irritated if I cannot use my mobile phone. |
| 7 | D4 | Utilizarea telefonului interferează cu relațiile mele sociale. | My phone use interferes with my social relationships. |
| 8 | D4 | Am primit critici legate de timpul petrecut pe telefon. | I have been criticised about the amount of time I spend on my phone. |
| 9 | D1 | Am încercat să reduc timpul petrecut pe telefon, dar nu am reușit. | I have tried to reduce the time I spend on my phone, but I have not succeeded. |
| 10 | D5 | Folosesc telefonul pentru a evita sarcinile neplăcute. | I use my phone to avoid unpleasant tasks. |
| 11 | D3 | Îmi pierd somnul din cauza utilizării excesive a telefonului. | I lose sleep because of my excessive phone use. |
| 12 | D1 | Mă simt vinovat(ă) după ce petrec mult timp pe telefon. | I feel guilty after spending a lot of time on my phone. |
| 13 | D1 | Nu pot rezista impulsului de a-mi verifica telefonul. | I cannot resist the urge to check my phone. |
| 14 | D2 | Mă simt dependent(ă) de telefonul mobil. | I feel dependent on my mobile phone. |
| 15 | D3 | Prioritizez telefonul față de alte responsabilități zilnice. | I prioritise my phone over other daily responsibilities. |
| 16 | D2 | Mă simt stresat(ă) când nu am telefonul aproape. | I feel stressed when my phone is not near me. |
| 17 | D1 | Folosesc telefonul chiar și în locuri sau momente nepotrivite. | I use my phone even in inappropriate places or at inappropriate times. |
| 18 | D3 | Utilizarea telefonului îmi afectează productivitatea. | My phone use affects my productivity. |
| 19 | D3 | Telefonul îmi afectează viața profesională sau academică. | My phone affects my professional or academic life. |
| 20 | D2 | Mă simt anxios/anxioasă fără telefonul mobil. | I feel anxious without my mobile phone. |
| 21 | D4 | Îmi neglijez familia din cauza timpului petrecut pe telefon. | I neglect my family because of the time I spend on my phone. |
| 22 | D3 | Mi s-a întâmplat să întârzii din cauza utilizării telefonului. | I have been late because of my phone use. |
| 23 | D4 | Prefer să stau pe telefon în loc să socializez în realitate. | I prefer to be on my phone rather than socialise in person. |
| 24 | D5 | Am tendința să folosesc telefonul când sunt trist(ă) sau stresat(ă). | I tend to use my phone when I feel sad or stressed. |
| 25 | D2 | Nu pot imagina o zi fără telefonul meu. | I cannot imagine a day without my phone. |
| 26 | D5 | Mă simt relaxat(ă) doar când folosesc telefonul. | I feel relaxed only when I am using my phone. |
| 27 | D5 | Am nevoie de telefon ca să mă simt conectat(ă) cu ceilalți. | I need my phone in order to feel connected to others. |

*Note.* D1, compulsive use and loss of control; D2, psychological dependence; D3, functional interference; D4, impairment of social functioning; D5, compensatory use. The five domains are content categories defined a priori for item development and are not empirically derived factors or validated subscales (see Sections 2.4.1 and 3.3 of the main text). All items were rated on a five-point Likert scale (1 = not at all, 5 = always) and were worded in the same direction; there were no reverse-scored items.

**Supplementary Table S2.** Academic procrastination index: item wording, descriptive statistics, and corrected item–total correlations (*N* = 973).

| **No.** | **Item (Romanian)** | **Item (English)** | ***M*** | ***SD*** | **CITC** |
| --- | --- | --- | --- | --- | --- |
| 1 | Scrierea unui referat/eseu | Writing a term paper/essay | 3.17 | 1.24 | .55 |
| 2 | Învățarea pentru un examen | Studying for an exam | 3.21 | 1.21 | .64 |
| 3 | Citirea bibliografiei recomandate | Reading the recommended bibliography | 3.15 | 1.21 | .27 |
| 4 | Participarea la cursuri/seminarii | Attending lectures/seminars | 2.86 | 1.32 | .61 |
| 5 | Îndeplinirea proiectelor de grup | Completing group projects | 2.98 | 1.45 | .67 |
| 6 | Înscrierea la examene/alte formalități | Registering for exams/other administrative tasks | 2.74 | 1.51 | .64 |

*Note.* *M*, mean; *SD*, standard deviation; CITC, corrected item–total correlation. Items were rated on a five-point Likert scale (1 = never, 5 = always); the score used in the analyses was the mean of the six items. Item 3 showed the lowest corrected item–total correlation and the lowest loading in the one-factor confirmatory model (.31 versus .63–.83); it was retained to preserve content coverage, and sensitivity analyses with a five-item score produced equivalent results (Sections 3.4 and 3.12 of the main text). The index is a conceptual adaptation and is not a translation of the Procrastination Assessment Scale for Students.

**Supplementary Table S3.** Self-reported reasons for procrastination: item wording, descriptive statistics, corrected item–total correlations, and correlations with academic procrastination and problematic smartphone use (*N* = 973).

| **No.** | **Reason (Romanian)** | **Reason (English)** | **Dim.** | ***M*** | ***SD*** | **% ≥ 4** | **CITC** | ***r* with AP** | ***r* with PSU** |
| --- | --- | --- | --- | --- | --- | --- | --- | --- | --- |
| 1 | Perfecționism (vreau să fie totul perfect înainte să încep) | Perfectionism (I want everything to be perfect before I start) | F2 | 3.49 | 1.25 | 51.0 | .07 | .15 [.09, .21]*** | .08 [.01, .14]* |
| 2 | Teamă de eșec | Fear of failure | F2 | 3.31 | 1.34 | 47.4 | .39 | .17 [.10, .23]*** | .34 [.29, .40]*** |
| 3 | Lipsă de interes | Lack of interest | F1 | 2.58 | 1.19 | 21.5 | .45 | .22 [.16, .28]*** | .29 [.23, .34]*** |
| 4 | Probleme de organizare | Difficulties with organisation | F1 | 2.66 | 1.21 | 24.3 | .51 | .15 [.08, .21]*** | .32 [.26, .37]*** |
| 5 | Distragere ușoară de la alte activități | Being easily distracted by other activities | F1 | 2.87 | 1.19 | 29.6 | .60 | .20 [.14, .26]*** | .47 [.42, .52]*** |
| 6 | Lipsă de energie/motivație | Lack of energy/motivation | F1 | 3.13 | 1.25 | 39.3 | .62 | .09 [.03, .16]** | .42 [.36, .47]*** |
| 7 | Nu-mi place sarcina | I dislike the task | F1 | 3.00 | 1.24 | 33.9 | .53 | .10 [.04, .16]** | .31 [.25, .37]*** |
| 8 | Subestimarea timpului necesar | Underestimating the time required | F1 | 3.00 | 1.20 | 33.2 | .59 | .23 [.17, .29]*** | .36 [.31, .42]*** |

*Note.* AP, academic procrastination; PSU, problematic smartphone use; Dim., dimension recovered by parallel analysis and principal-axis factoring with Promax rotation; *M*, mean; *SD*, standard deviation; CITC, corrected item–total correlation computed against the total score across the eight reasons; % ≥ 4, percentage of participants selecting 4 or 5 on the five-point scale. Items were rated from 1 (not at all true) to 5 (very true). F1, task-aversion and self-regulation-difficulty dimension (six items; α = .82); F2, fear-of-failure and perfectionism dimension (two items); the two dimensions were weakly correlated (Φ = .22). At the dimension level, F1 was associated with problematic smartphone use, *r* = .50, 95% CI [.45, .54], and negatively with general self-efficacy, *r* = −.12, 95% CI [−.19, −.06], whereas F2 showed a weaker association with problematic use, *r* = .26, 95% CI [.20, .32], and a positive association with self-efficacy, *r* = .12, *p* < .001; entered together with problematic smartphone use in the prediction of academic procrastination, the two dimensions explained Δ*R*² = .038, *F*(2, 969) = 19.91, *p* < .001. Item-level correlations were computed with the six-item academic procrastination score and the 27-item global MSUPS score; values in brackets are 95% confidence intervals based on Fisher’s *z* transformation. Because these analyses were exploratory, significance thresholds were not adjusted for multiple comparisons. **p* < .05. ***p* < .01. ****p* < .001.

**Supplementary Table S4.** Informed consent form, Romanian and English.

| **Section** | **Text (Romanian)** | **Text (English)** |
| --- | --- | --- |
| Scopul studiului / Purpose of the study | Sunteți invitat(ă) să participați la un studiu științific care are ca scop identificarea impactului utilizării telefonului mobil asupra procrastinării la studenți. | You are invited to take part in a scientific study whose purpose is to identify the impact of mobile phone use on procrastination in university students. |
| Colectarea datelor / Data collection | Participarea este voluntară, iar datele oferite (răspunsuri la chestionare și informații socio-demografice) vor fi colectate prin Google Forms, în mod anonim și confidențial, conform Regulamentului (UE) 2016/679 (GDPR). | Participation is voluntary, and the data provided (questionnaire responses and socio-demographic information) will be collected through Google Forms, anonymously and confidentially, in accordance with Regulation (EU) 2016/679 (GDPR). |
| Caracterul voluntar / Voluntary participation | Participarea dumneavoastră la această cercetare este voluntară, având posibilitatea să vă retrageți din studiu în orice moment al completării chestionarului, iar acest lucru nu vă va afecta în niciun fel. De asemenea, pentru participarea la acest studiu nu sunt acordate beneficii materiale, însă, dacă în continuare doriți să participați, veți aduce contribuții importante din punct de vedere științific în domeniul educațional. | Your participation in this research is voluntary; you may withdraw from the study at any point while completing the questionnaire, and this will not affect you in any way. No material benefits are provided for taking part in this study; however, if you still wish to participate, you will make an important scientific contribution to the field of education. |
| Confidențialitate / Confidentiality | Dacă decizia dumneavoastră de a participa la această cercetare este încă valabilă, vă rog să semnați consimțământul informat pe care îl regăsiți imediat după citirea acestui text și să completați chestionarul din formularul Google pentru care ați primit linkul de acces. Toate informațiile pe care le furnizați prin participarea la această cercetare sunt strict confidențiale, în conformitate cu regulile Universității de Medicină, Farmacie, Științe și Tehnologie „George Emil Palade” din Târgu Mureș. Informațiile vor fi introduse într-o bază de date stocată pe un laptop la care accesul se realizează doar în baza unei parole. Totodată, informațiile vor fi codificate, asigurându-se toate condițiile pentru respectarea confidențialității. | If your decision to take part in this research still stands, please sign the informed consent form provided immediately after this text and complete the questionnaire in the Google Form for which you received the access link. All information you provide by participating in this research is strictly confidential, in accordance with the regulations of the "George Emil Palade" University of Medicine, Pharmacy, Science and Technology of Târgu Mureș. The information will be entered into a database stored on a password-protected laptop. The information will also be coded, so that all conditions for maintaining confidentiality are met. |
| Prelucrarea datelor / Data processing | Conform cerințelor Regulamentului (UE) 2016/679 privind protecția persoanelor fizice în ceea ce privește prelucrarea datelor cu caracter personal și privind libera circulație a acestor date și de abrogare a Directivei 95/46/CE (Regulamentul general privind protecția datelor) și ale Legii nr. 506/2004 privind prelucrarea datelor cu caracter personal și protecția vieții private, am obligația de a administra în condiții de siguranță și numai pentru scopurile specificate datele pe care le veți furniza: date socio-demografice și răspunsuri subiective la chestionare. | In accordance with the requirements of Regulation (EU) 2016/679 on the protection of natural persons with regard to the processing of personal data and on the free movement of such data, and repealing Directive 95/46/EC (General Data Protection Regulation), and of Law no. 506/2004 on the processing of personal data and the protection of privacy, I am required to process securely and only for the stated purposes the data you provide: socio-demographic data and subjective responses to the questionnaires. |
| Exprimarea consimțământului / Expression of consent | Consimțământul informat va fi exprimat prin următoarea sintagmă: „Am luat la cunoștință Informarea și consimt ca datele mele cu caracter personal să fie prelucrate, în conformitate cu legislația europeană – Regulamentul (UE) 2016/679 privind protecția și prelucrarea datelor cu caracter personal.” Această sintagmă se află la începutul chestionarului, iar acordul privind consimțământul informat se ia în considerare prin bifarea casetei corespunzătoare răspunsului „DA”. | Informed consent will be expressed through the following statement: "I have read and understood the Information Notice and I consent to my personal data being processed in accordance with European legislation – Regulation (EU) 2016/679 on the protection and processing of personal data." This statement appears at the beginning of the questionnaire, and consent is recorded by ticking the box corresponding to the answer "YES". |
| Item de consimțământ / Consent item | ☐ Da, sunt de acord să particip la acest studiu de cercetare și confirm că am peste 18 ani. | ☐ Yes, I agree to take part in this research study and I confirm that I am over 18 years of age. |

*Note.* The informed consent text was displayed in full on the first page of the Google Form, before access to the questionnaire items. Participation was conditional upon ticking the agreement box.

**Supplementary Table S5.** Socio-demographic grid, Romanian and English.

| **No.** | **Variable** | **Item and response options (Romanian)** | **Item and response options (English)** |
| --- | --- | --- | --- |
| 1 | Gen / Gender | Gen: ☐ Feminin ☐ Masculin ☐ Altele | Gender: ☐ Female ☐ Male ☐ Other |
| 2 | Instituția de învățământ / Educational institution | Facultatea / Școala: ____________________ | Faculty / School: ____________________ |
| 3 | An de studiu / Year of study | Anul de studiu: ☐ I ☐ II ☐ III ☐ IV ☐ V ☐ Elev (liceu) | Year of study: ☐ I ☐ II ☐ III ☐ IV ☐ V ☐ Secondary-school pupil |
| 4 | Vârstă / Age | Vârsta: ____________________ | Age: ____________________ |
| 5 | Utilizare zilnică / Daily use | Câte ore pe zi utilizezi telefonul mobil în timpul săptămânii? ☐ mai puțin de o oră ☐ între o oră și 2 ore ☐ între 2 și 3 ore ☐ între 3 și 4 ore ☐ între 4 și 5 ore ☐ peste 5 ore | How many hours a day do you use your mobile phone during the week? ☐ less than one hour ☐ between one and 2 hours ☐ between 2 and 3 hours ☐ between 3 and 4 hours ☐ between 4 and 5 hours ☐ more than 5 hours |
| 6 | Rang în fratrie / Sibling position | Care este locul tău în fratrie? ____________________ | What is your position among siblings? ____________________ |
| 7 | Vârsta primului telefon mobil / Age at first mobile phone ownership | La ce vârstă ai avut primul tău telefon mobil? ☐ înainte de 8 ani ☐ între 8 și 10 ani ☐ după 11 ani ☐ după 12 ani ☐ după 13 ani ☐ după 14 ani | At what age did you have your first mobile phone? ☐ before 8 years ☐ between 8 and 10 years ☐ after 11 years ☐ after 12 years ☐ after 13 years ☐ after 14 years |

*Note.* The socio-demographic grid was administered immediately after the informed consent and before the MSUPS and procrastination items. No directly identifying data (name, e-mail address, IP address) were collected. Age was recorded in categories and recoded to representative values for secondary analyses only; daily use categories were recoded to interval midpoints (0.5–5.5 hours) for parametric analyses (Section 2.4.4 of the main text).
