# Supplementary material for: Problematic smartphone use is more strongly associated with academic procrastination than self-reported daily use duration
Source: Front Psychol. 2026 Oct 28;17:1963771. doi: 10.3389/fpsyg.2026.1963771 (PMC13752132; doi:10.3389/fpsyg.2026.1963771)
Supplement: Supplementary file 2 [file Supplementary_file_2.docx]

**Supplementary Material**

**Supplementary Table S6. Age at first mobile phone ownership and current daily smartphone use (post hoc analysis).**

Age at first mobile phone ownership (six ordered categories) was examined in relation to current daily smartphone use (hours, category midpoints; Section 2.4.4 of the main text), using Spearman’s rank-order correlation and linear regression with HC3 robust standard errors adjusted for current age category. The analysis was added after the main analyses were completed and is reported as post hoc, without adjustment for multiple comparisons.

| **Analysis** | **Coefficient** | **SE (HC3)** | **p** | **95% CI** | **n** |
| --- | --- | --- | --- | --- | --- |
| Spearman’s rank-order correlation | ρ = −.117 | — | < .001 | — | 972 |
| Regression: age at first ownership | b = −0.117 | 0.035 | < .001 | [−0.186, −0.048] | 970 |
| Regression: current age category | b = −0.020 | — | .258 | — | 970 |

Note. b, unstandardized regression coefficient. Age at first mobile phone ownership was coded in ascending order of age, so negative coefficients indicate that participants who received their first mobile phone later reported slightly shorter current daily use. Model R² = .020. The effect is small; age at first ownership was assessed retrospectively in broad categories whose wording does not make them mutually exclusive, and the cross-sectional design permits no causal inference.
